# Battered woman syndrome and PTSD in women who kill their abusing partner: a study in medical jurisprudence

**DOI:** 10.1192/bjo.2021.708

**Published:** 2021-06-18

**Authors:** Sharmilaa Lagunathan

**Affiliations:** Leicester Medical School, University of Leicester

## Abstract

**Aims:**

The aim of the study was to identify any symptoms or features of Battered Woman Syndrome (BWS) or Post-traumatic Stress Disorder (PTSD) that may be associated with, or explain, abused women killing their abuser; and the extent to which such identified symptoms or features have been deemed, or are potentially relevant, to past and now reformed partial defences to murder in English law. Hence two sub-studies were completed.

**Method:**

The first sub-study identified mental symptoms of BWS or PTSD apparent in battered women who kill their abuser; achieved by identifying relevant research papers, through applying a ‘rapid review’ approach to three databases: PubMed, PsychInfo and PsychArticles. The second sub-study identified by legal research reported Court of Appeal (CA) judgments on women appealing their conviction of the murder of their abusive partner. It then analysed the legal approach taken towards evidence of the effects of abuse upon these women before and after relevant statutory law reform (although no CA cases were identified post-reform).

**Result:**

The first sub-study identified and reviewed six symptoms or features, within three quantitative and three qualitative studies, that appeared to be associated with, or described by, abused women killing their abuser. These included helplessness, symptoms associated with PTSD, plus fear, isolation, experience of escalation of violence and cycle of violence. From the CA cases the perpetrators of killings that occurred prior to 04.10.2010 (the date of law reform) were usually successful in having their conviction overturned based upon diminished responsibility; but not provocation, because of the requirement of ‘sudden loss of self control’. ‘Loss of control’, which replaced provocation, appears highly likely to be capable of reducing murder to manslaughter based upon symptoms of BWS, or PTSD. However, the amended defence of diminished responsibility is likely to exclude evidence of BWS, but allow evidence of PTSD, because of its requirement of the defendant suffering from ‘a recognised medical condition’.

**Conclusion:**

This study demonstrated particular symptoms or features of BWS or PTSD associated with abused women killing their abusers plus their very different relevance to two partial defences to murder, pre and post law reform.

